# Further Optimization of the mGlu_1_ PAM VU6024578/BI02982816: Discovery and Characterization of VU6033685

**DOI:** 10.1021/acschemneuro.5c00014

**Published:** 2025-02-05

**Authors:** Carson W. Reed, Jacob F. Kalbfleisch, Jeremy A. Turkett, Trevor A. Trombley, Paul K. Spearing, Daniel H. Haymer, Marc Quitalig, Jonathan W. Dickerson, Daniel J. Foster, Annie L. Blobaum, Olivier Boutaud, Hyekyung P. Cho, Colleen M. Niswender, Jerri M. Rook, Henning Priepke, Heiko Sommer, Stefan Scheuerer, Daniel Ursu, P. Jeffrey Conn, Bruce J. Melancon, Craig W. Lindsley

**Affiliations:** † Warren Center for Neuroscience Drug Discovery, Vanderbilt University, Nashville, Tennessee 37232, United States; ‡ Department of Pharmacology, Vanderbilt University School of Medicine, Nashville, Tennessee 37232, United States; § Department of Chemistry, Vanderbilt University, Nashville, Tennessee 37232, United States; ∥ Vanderbilt Kennedy Center, Vanderbilt University Medical Center, Nashville, Tennessee 37232, United States; ⊥ Vanderbilt Brain Institute, Vanderbilt University, Nashville, Tennessee 37232, United States; # Vanderbilt Institute of Chemical Biology, Vanderbilt University, Nashville, Tennessee 37232, United States; ∇ Boehringer Ingelheim Pharma GmbH & Co. KG, Birkendorfer Str. 65, 88397 Biberach Germany

**Keywords:** metabotropic glutamate receptor subtype 1 (mGlu_1_), positive allosteric modulator (PAM), cognition, metabolism, amphetamine-induced hyperlocomotion, pharmacokinetics

## Abstract

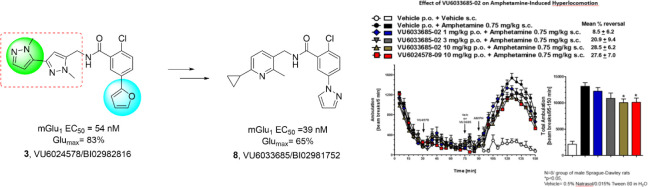

Herein, we report the further chemical optimization of the metabotropic glutamate receptor subtype 1 (mGlu_1_) positive allosteric modulator (PAM) VU6024578/BI02982816 and the discovery of VU6033685/BI1752. PAM VU6033685/BI1752 was developed through an iterative process wherein, after the furanyl moiety (a potential toxicophore) was replaced by an *N*-linked pyrazole, a diversity screen identified a quinoline core, which was further truncated to a pyridine scaffold. PAM VU6033685/BI1752 proved to be a potent and selective mGlu_1_ PAM with efficacy in both amphetamine-induced hyperlocomotion (AHL) and novel object recognition (NOR) with a clear pharmacokinetic–pharmacodynamic (PK/PD) relationship. VU6024578/BI02982816 was efficacious and well tolerated in rats but not dogs, whereas VU6033685/BI1752 elicited adverse events (AEs) in both rats and dogs. These AEs, noted in two distinct mGlu_1_ PAM chemotypes, cast a shadow on an otherwise promising molecular target to address multiple symptom clusters in schizophrenic patients.

## Introduction

There is a renaissance in CNS drug discovery, and psychiatry in particular, with a quest for fundamentally new targets for the treatment of schizophrenia.^1−5^ Driven by the approval of Cobenfy (KarXT), an M_1_/M_4_ preferring agonist, and the first new mechanism (muscarinic activation) for schizophrenia in decades,^6−8^ there is a focus on nondopaminergic targets. Following on the heels of Cobenfy comes Cerevel’s Emraclidine, a selective M_4_ PAM, recently acquired by AbbVie.^9^ Decades of work on M_4_ PAMs in our laboratories have demonstrated that the antipsychotic effects of M_4_ activation, and the ability to inhibit dopamine release, are dependent on coactivation of the metabotropic glutamate receptor subtype 1 (mGlu_1_); additionally, M_4_ PAM activity can be blocked by mGlu_1_ NAMs.^10^ These data led our group to develop mGlu_1_ PAMs as a complementary therapeutic strategy to M_4_ PAMs.^11−13^ Second-generation mGlu_1_ PAM tool compounds reversed psychostimulant-induced hyperlocomotion, displayed efficacy in novel object recognition, and improved cognitive performance in a subchronic phencyclidine (PCP) NMDA hypofunction model. Moreover, we demonstrated that mGlu_1_ PAMs inhibit dopamine release in an endocannabinoid-dependent manner, as do M_4_ PAMs.^11−13^ Human genetic data also support mGlu_1_ as a viable target for schizophrenia, with numerous loss-of-function single nucleotide polymorphisms (SNPs) in *GRM1*, the gene-encoding mGlu_1_ in schizophrenia and bipolar patients. Encouragingly, mGlu_1_ PAMs can rescue signaling of these mutants *in vitro*.^14^

**Figure 1:**
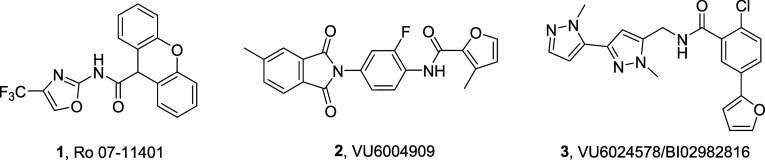
Structures of exemplar *in vitro* and *in vivo* mGlu_1_ PAM tool compounds **1**–**3**.

Existing mGlu_1_ PAM tool compounds **1** and **2** (Figure 1) were acceptable for *in vivo* target validation studies, but they lacked drug-like profiles to advance.^15−24^ Hence, we recently reported on a novel mGlu_1_ PAM, VU6024578/BI02982816 (compound **3**) that displayed robust efficacy in rodent models of antipsychotic-like activity and cognition, with a clear PK/PD relationship and a path forward with a biomarker strategy.^25^ However, the naked furanyl moiety, as a potential toxicophore and unexpected adverse events (AEs) observed in dogs, led to the termination of **3**. Were the AEs chemotype-driven, or could they be due to unfavorable signal bias or activity at an mGlu_1_/mGlu_5_ heterodimer? Here, we describe a lead optimization campaign that identified a replacement for the undesired furanyl moiety and resulted in the discovery of a fundamentally new chemotype, exemplified by VU6033685/BI1752. With a new mGlu_1_ PAM in hand, we disclose the full characterization (molecular pharmacology, *in vitro* and *in vivo* DMPK, and behavioral) of VU6033685/BI1752, as well as more egregious AEs in both rats and dogs, casting a shadow on a promising schizophrenia target.

## Results and Discussion

### Chemical Lead Optimization

**Figure 2:**
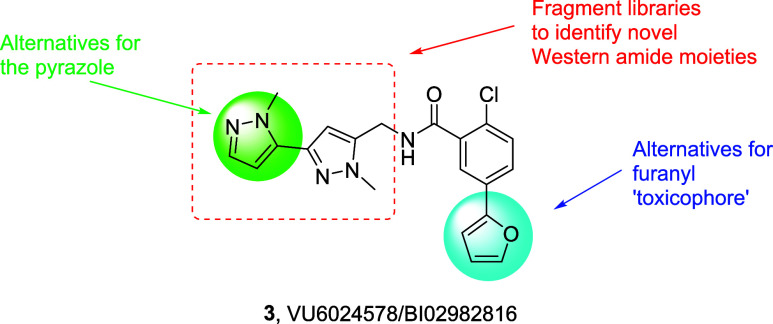
Envisioned, multidimensional chemical optimization plan for mGlu_1_ PAM **3**.

**Figure 3:**
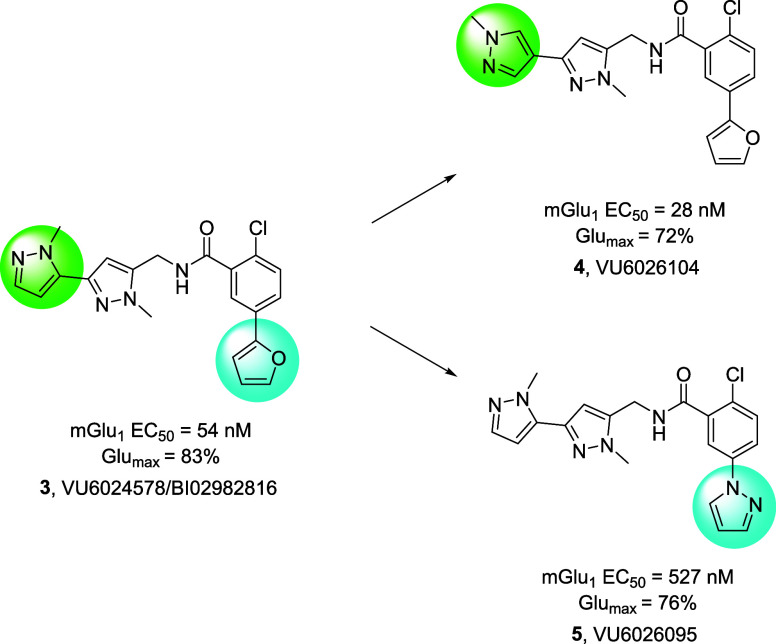
Chemical optimization plan for mGlu_1_ PAM **3**, leading to the identification of novel pyrazole regioisomer **4** (EC_50_ = 28 nM, 72% Glu Max) and *N*-linked pyrazole **5** (EC_50_ = 527 nM, 76% Glu Max) as an alternative for the furanyl ring.

The chemical optimization plan for **3** was based on a multidimensional strategy (Figure 2) to ultimately develop a distinct chemotype from **3** to assess the AEs observed in dogs with VU6024578/BI02982816 to understand mechanism-based or chemotype-based effects.^25^ At the same time, we had to identify an alternative for the naked southern furanyl moiety, a known toxicophore.^26^ In parallel, efforts examined alternatives for the western pyrazole, as well as the furanyl moiety (Figure 3). SAR was steep when surveying alternatives for the western pyrazole, but regioisomeric **4** was identified with enhanced potency (human EC_50_ = 28 nM, 72% Glu Max) over **3**. PAM **4** was also potent on rat mGlu_1_ (EC_50_ = 37 nM, 137% Glu Max), with good unbound fraction in plasma (*f*_u_ (h, r) = 0.063, 0.040) and brain (*f*_u_ (rat) = 0.044), CNS penetration (*K*_p_ = 0.89, *K*_p,uu_ = 0.98), clean CYP_450_ profile (>30 μM at 3A4, 2D6 and 2C9; 14.7 μM at 1A2), and moderate predicted hepatic clearance (CL_hep_ (h, r) = 15.1 mL/min/kg and 34.2 mL/min/kg).^27^ However, these positive attributes did not address the liability of the furanyl ring. A broad survey of 5- and 6-membered heterocycles identified a single alternative for the furanyl ring, an *N*-linked pyrazole, **5**. While an order of magnitude less potent (human mGlu_1_ EC_50_ = 551 nM, rat mGlu_1_ EC_50_ = 558 nM, 105%) than **3**, the overall DMPK profile was far more attractive. PAM **5** displayed low predicted hepatic clearance (CL_hep_ (h, r) = 4.6 mL/min/kg and 21.4 mL/min/kg), high unbound fraction in plasma (*f*_u_ (h, r) = 0.143, 0.095) and brain (*f*_u_ (rat) = 0.185), and an exceptional CYP_450_ inhibition profile (>30 μM at 3A4, 2D6, 2C9, and 1A2). CNS penetration in rat (*K*_p_ = 0.3, *K*_p,uu_ = 0.52) was lower than **3**, but *in vivo* rat IV/PO PK was improved (CL_p_ = 2.9 mL/min/kg, *t*_1/2_ = 3 h, *V*_ss_ = 0.63 L/kg; 45.7% F).^27^ Thus, we elected to maintain the *N*-linked pyrazole in a fragment screening approach to identify alternative amides for the western *bis*-pyrazole moiety and hopefully improve the mGlu_1_ PAM functional potency.

**Figure 4:**
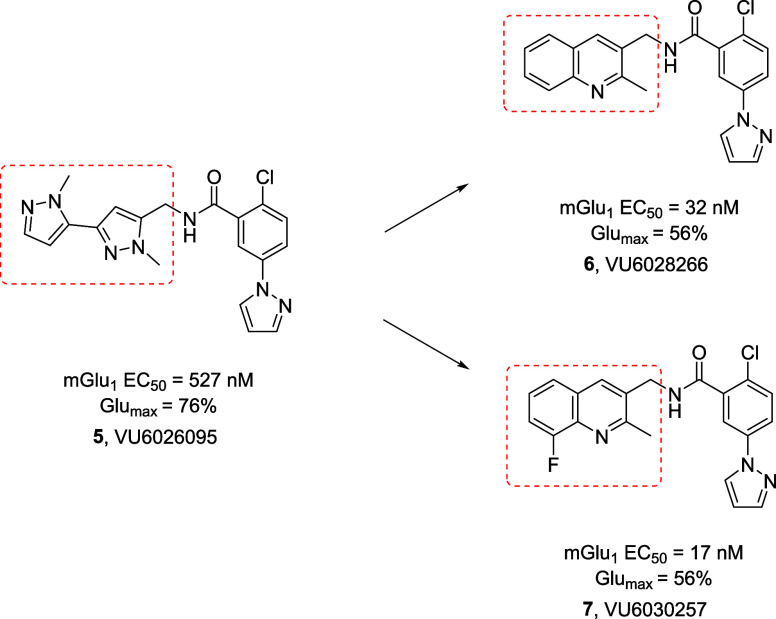
Fragment library amide scan for the chemical optimization of mGlu_1_ PAM **5**, leading to the identification of novel quinoline amides **6** (EC_50_ = 32 nM, 56% Glu Max) and **7** (EC_50_ = 17 nM, 56% Glu Max) as alternatives for bis-pyrazole ring system.

A large scan of various heteroaryl methyl amines and benzyl amines was coupled to the requisite *N*-pyrazole linked benzoic acid; however, the vast majority of analogs proved to be weak or inactive mGlu_1_ PAMs. Two quinoline-derived amide analogs, **6** (EC_50_ = 32 nM, 56% Glu Max) and **7** (EC_50_ = 17 nM, 56% Glu Max), proved to be very potent mGlu_1_ PAMs, albeit with modest efficacy (Figure 4). In the case of **7**, this represented an ∼31-fold increase in the mGlu_1_ PAM potency over **5**. While **6** was more potent than **5,** it possessed a poor *in vitro* DMPK profile (CL_hep_ (h, r) = 15.8 mL/min/kg and 47.8 mL/min/kg; plasma *f*_u_ (h, r) = 0.037, 0.054; brain *f*_u_ (r) = 0.047; CYP_450_ inhibition: (IC_50_s = 8.8 μM (3A4), 6.9 μM (2D6), 6.4 μM (2C9), and 15.9 μM (1A2)) and was clearly not advanceable.^27^ However, we wanted to examine *in vivo* rat IV/PO PK to fully assess this novel chemotype and establish if an *in vitro*:*in vivo* correlation (IVIVC) existed. For **6**, an attractive rat *in vivo* PK profile (CL_p_ = 15.3 mL/min/kg, *t*_1/2_ = 1.1 h, *V*_ss_ = 1.64 L/kg; 64% F, 30 min *T*_max_) was observed, but with a lack of IVIVC. Finally, this new chemotype was predicted to be CNS penetrant in man (P-gp, MDCK-MDR1 ER = 0.5, P_app_ = 39 × 10^–6^ cm/s). The analogous 8-fluoro congener **7** possessed even greater mGlu_1_ PAM activity (EC_50_ = 17 nM, 56% Glu Max), and we hoped that the electronegative fluorine might improve the CYP_450_ profile. Unfortunately, the 8-F moiety had limited impact on the CYP450 profile (IC_50_s = 9.0 μM (3A4), > 30 μM (2D6), 5.9 μM (2C9), and 11.2 μM (1A2)), and the *in vitro* disposition (CL_hep_ (h, r) = 13.3 mL/min/kg and 46.8 mL/min/kg; plasma *f*_u_ (h, r) = 0.053, 0.016; brain *f*_u_ (r) = 0.040) was similar to **6**. However, the rat *in vivo* profile did improve (CL_p_ = 8 mL/min/kg, *t*_1/2_ = 2.1 h, *V*_ss_ = 1.0 L/kg, and K_p_ = 0.98).^27^ Thus, while not optimal, the effort to identify a chemically distinct mGlu_1_ PAM is moving in the right direction.

**Figure 5:**
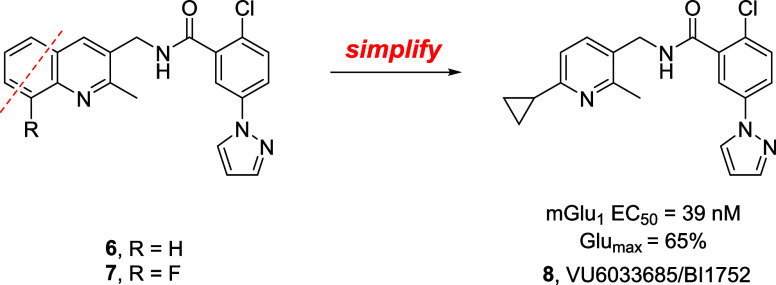
Truncation and simplification of quinoline amides **6** and **7** to a pyridine core provided **8** (EC_50_ = 32 nM, 56% Glu Max), a chemically distinct mGlu_1_ PAM for further profiling.

Having experienced DMPK challenges with quinolines in the past, we elected to truncate the quinoline core to a pyridine core and surveyed a variety of pyridyl methyl amides (Figure 5). This exercise proved highly effective, leading to the discovery of PAM **8** (EC_50_ = 39 nM, 65% Glu Max), which addressed the *in vitro* and *in vivo* DMPK liabilities (CYP450 profile, predicted hepatic clearance, protein binding, etc.) of **6** and **7** and appeared advanceable. The potency of **8** represented an ∼14-fold improvement over **5** and replaced both the furanyl moiety as well as the *bis*-pyrazole ring system, affording a chemically distinct mGlu_1_ PAM for in-depth profiling.

### Chemical Synthesis

**Scheme 1:**
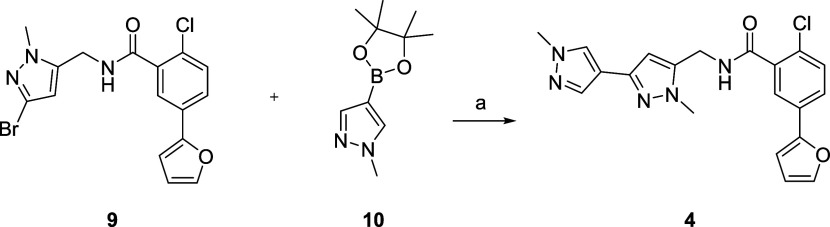
Synthesis of VU6026104 (**4**) Reagents and conditions. (a) Pd(dppf)Cl_2_, Na_2_CO_3_, 1,4-dioxane:H_2_O (3:1), 100°C, 2 h, 10%.

The syntheses of novel mGlu_1_ PAMs **4**–**8** were straightforward and employed readily available starting materials. For the synthesis of the pyrazole regioisomer **4** (Scheme 1), we employed an advanced intermediate **9**, previously described in the synthesis of **3**, and performed a Suzuki coupling with boronic ester **10**.^27^ While the yield was low (10%), this afforded sufficient material for evaluation.

**Scheme 2:**
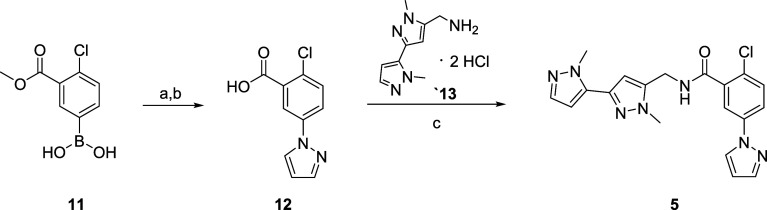
Synthesis of VU6026095 (**5**) Reagents and conditions. (a) Cu(OAc)_2_, pyrazole, pyridine, DMF, 70°C, 2h, 58%; (b) NaOH, THF:MeOH:H_2_O, rt, 18 h, 89%; (c) HATU, DIEA, DMF, 0°C to rt, 28%.

For the synthesis of the *N*-linked pyrazole **5** (Scheme 2), we utilized commercial boronic acid **11** in a copper-catalyzed Chan–Lam coupling^28^ with 1*H*-pyrazole. This led to, after saponification, the production of *N*-linked pyrazole **12** in 52% yield for the two steps. Finally, a HATU-mediated amide coupling with the previously described *bis*-pyrazole amine **13** delivered **5** in 28% yield.^27^

**Scheme 3:**
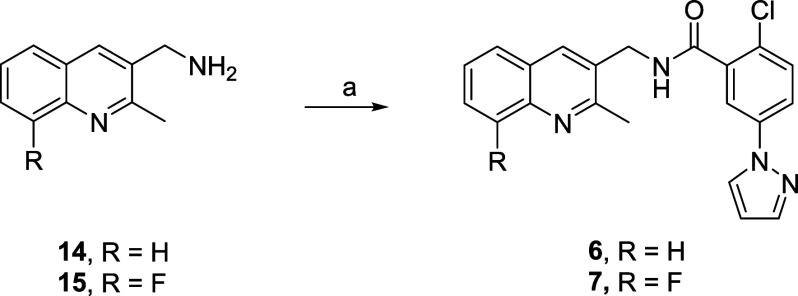
Synthesis of VU6028266 (**6**) and VU6030257 (**7**) Reagents and conditions. (a) **12**, HATU, DIEA, DMF, 0°C to rt, 43% for **6** and 37% for **7**.

**Scheme 4:**
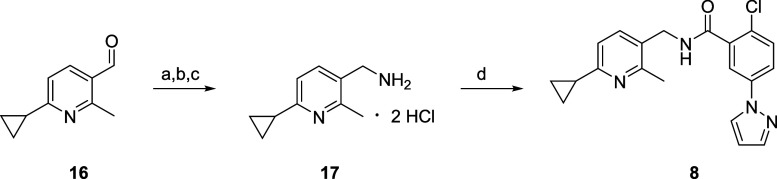
Synthesis of VU6033685/BI1752 (**8**) Reagents and conditions. (a) NH_2_OH·HCl, NaOAc, EtOH, rt, 2 h; (b) Zn, AcOH, rt, 4 h; (c) HCl, 92% over three steps; (d) **12**, HATU, DIEA, DMF, 0°C to rt, 67%.

Quinoline analogs **6** and **7** were prepared in a single step (Scheme 3) from commercial amines **14** and **15**, via a HATU-mediated amide coupling reaction with **12** to provide **6** and **7**, respectively, in yields of ∼40%. Finally, mGlu_1_ PAM **8** was prepared according to Scheme 4. Starting from commercial aldehyde **16**, conversion to the corresponding oxime, followed by Zn-mediated reduction and conversion to the *bis*-HCl salt afforded the pyridyl methyl amine **17** in 92% yield over the three steps. Then, a HATU-mediated coupling reaction between **17** and acid **12** gave **8** in 67% isolated yield.^27^

### Molecular Pharmacology and DMPK Profile of 8

The novel mGlu_1_ PAM **8** was a potent PAM across species (human EC_50_ = 39 nM, 65% (*n*= 9); rat EC_50_ = 107 nM, 109% Glu Max (*n* =7); mouse EC_50_ = 52 nM, 102% (*n*= 3); dog EC_50_ = 93 nM, 79% (*n* = 3)) and selective (>10 μM at mGlu_2–4, 7,8_) while a very weak mGlu_5_ PAM (EC_50_ = 3,760 nM, 72%).^27^ From our experience with mGlu_5_ PAMs, this was a potency value that would not interfere with evaluating selective mGlu_1_ activation.^29^ In terms of physicochemical properties (Table 1), PAM **8** displayed acceptable solubility (>100 μM @ pH2.2, < 1.0 μM @pH6.8, FASSGF (572 μg/mL) and FASSIF (23.6 μg/mL)) at neutral pH and exceptional solubility under acidic conditions. Unlike **4**-**7**, PAM **8** demonstrated an attractive, and advanceable, *in vitro* DMPK profile (CL_hep_ (h, r, d, c) = 9.9 mL/min/kg, 46.7 mL/min/kg, 15.1 mL/min/kg, 33.2 mL/min/kg; plasma *f*_u_ (h, r, d, c) = 0.063, 0.097, 0.099, 0.46; brain *f*_u_ (r) = 0.062 and CYP_450_ inhibition (IC_50_s = 23.8 μM (3A4), > 30 μM (2D6), 22.3 μM (2C9) and 26 μM (1A2)). PAM **8** was predicted to be highly CNS penetrant in humans, with MDCK-MDR1 ER = 0.93, Papp = 42 × 10^–6^ cm/s, and was found to be highly CNS penetrant in rats (K_p_ = 1.8; K_p,uu_ = 1.2). Rat *in vivo* PK was favorable (CL_p_ = 26.4 mL/min/kg, *t*_1/2_ = 2.1 h, *V*_ss_ = 5.1 L/kg; 42.8% F, 30 min *T*_max_).^27^ Thus far, the profile of **8** warranted further progression down the lead optimization flowchart and into behavioral pharmacology assessment.

**Table 1:**
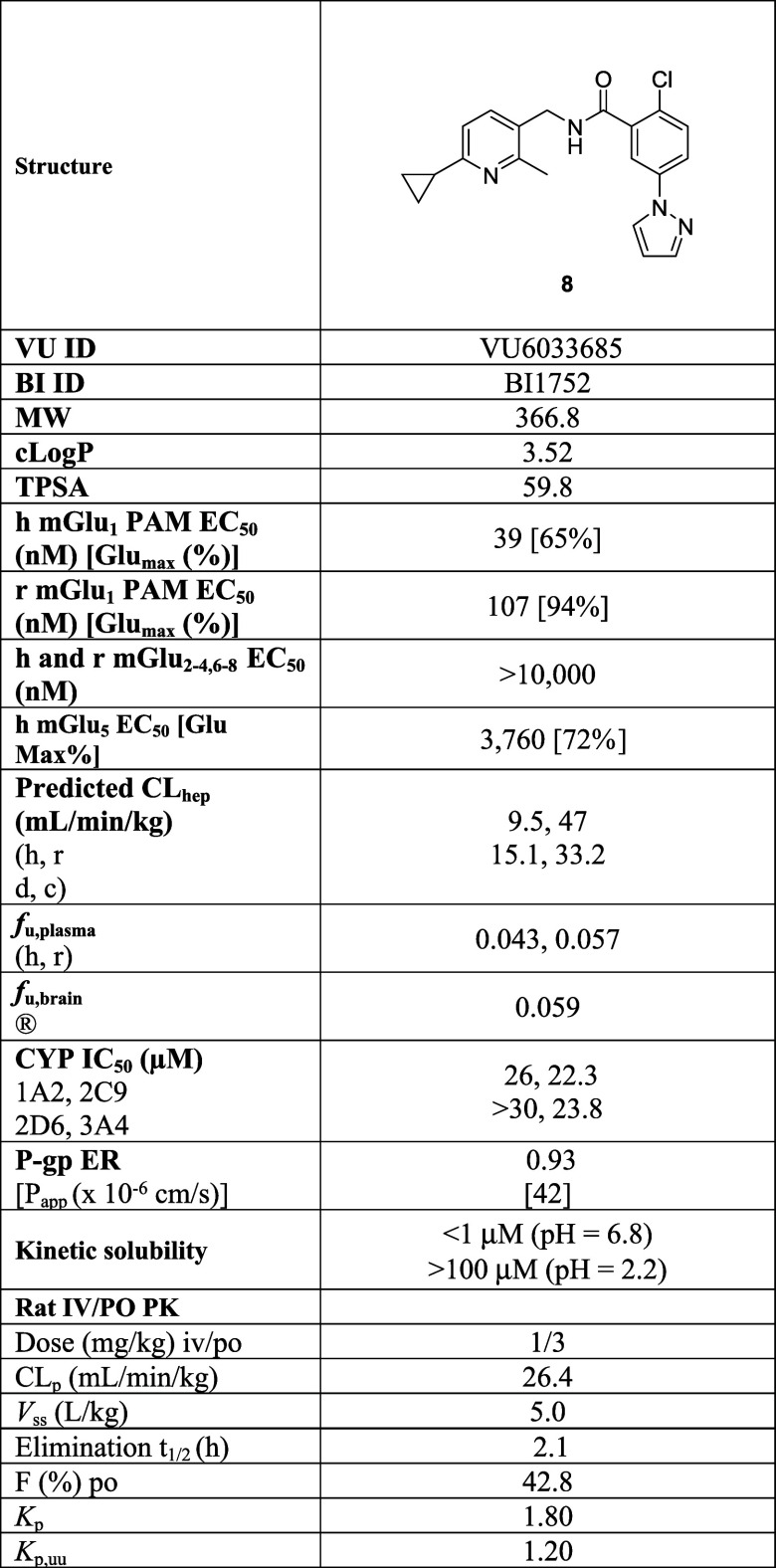
Pharmacology and *In Vitro* and *In Vivo* DMPK Profile of **8**

### Behavioral Pharmacology of 8

**Figure 6:**
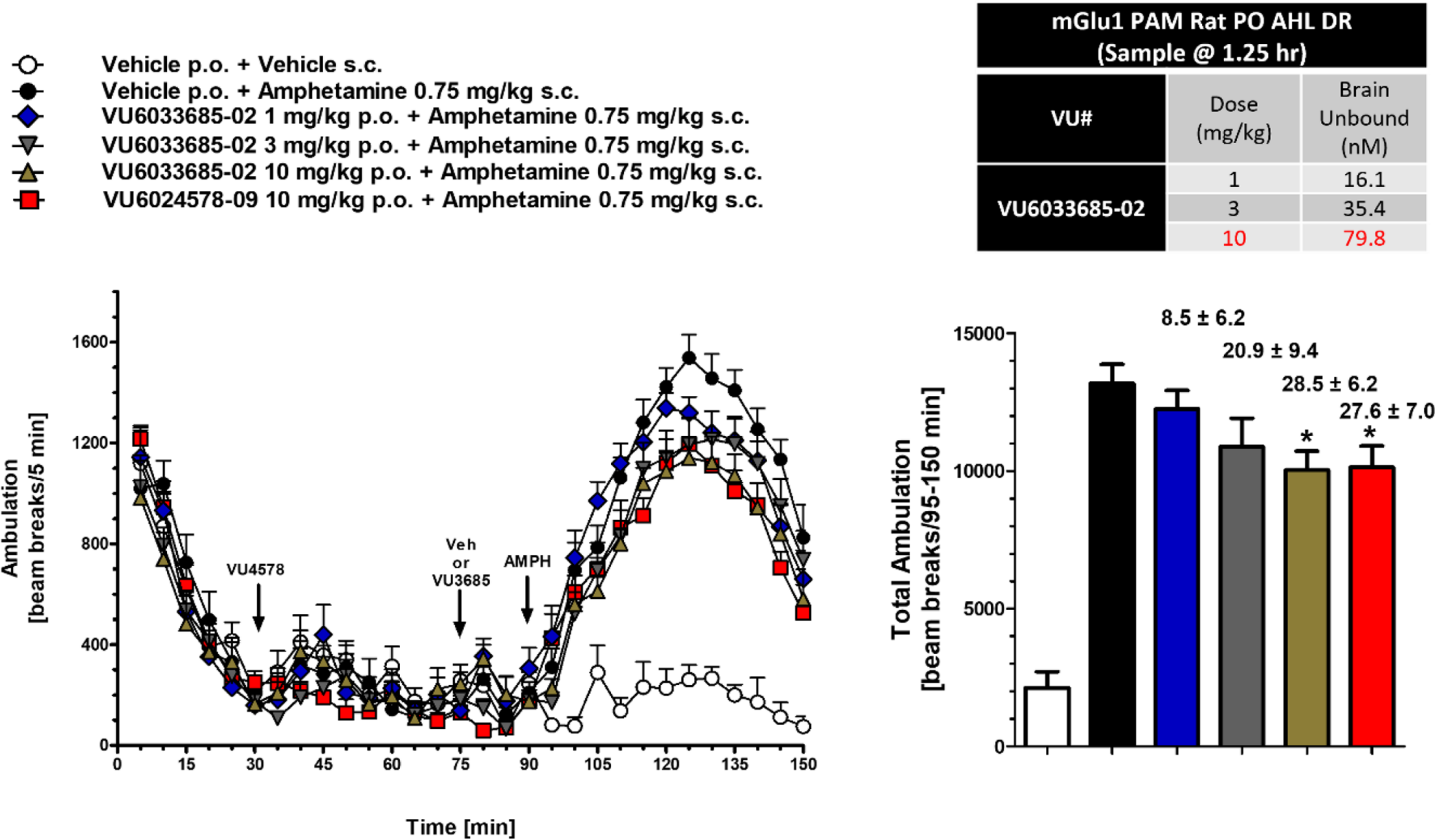
Rat amphetamine-induced hyperlocomotion and reversal by VU6033685 (**8**). Amphetamine (0.75 mg/kg SC) induced robust hyperlocomotion, which was dose-dependently reversed by oral administration (0.5% Natrasol/0.015% Tween 80) of **8**. A clear PK/PD relationship with efficacy was noted at ∼0.7-fold the *in vitro* rat mGlu_1_ EC_50_ in unbound brain, in agreement with the control, VU6024578 (**3**).

As PAM **3** reversed amphetamine-induced hyperlocomotion (AHL), a standard preclinical psychosis model where both M_4_ PAMs and clinically available antipsychotic drugs display robust efficacy; at unbound brain levels at ∼0.7-fold the rat mGlu_1_ EC_50_, we evaluated **8** in this paradigm employing **3** as a positive control (Figure 6).^25^ Here, administration of 0.75 mg/kg of amphetamine subcutaneously (SC) induced a robust hyperlocomotive state in rats (>1800 beam breaks), which was dose-dependently reversed by oral administration of **8**. Satellite rat PK taken at the 2.5 h end of study time point showed that at the 10 mg/kg minimum effective dose (MED), there was a free brain concentration of 79.8 nM (∼0.74-fold the *in vitro* EC_50_ of 107 nM). Moreover, PAM **8** was of comparable efficacy to the standard **3**.^27^

**Figure 7:**
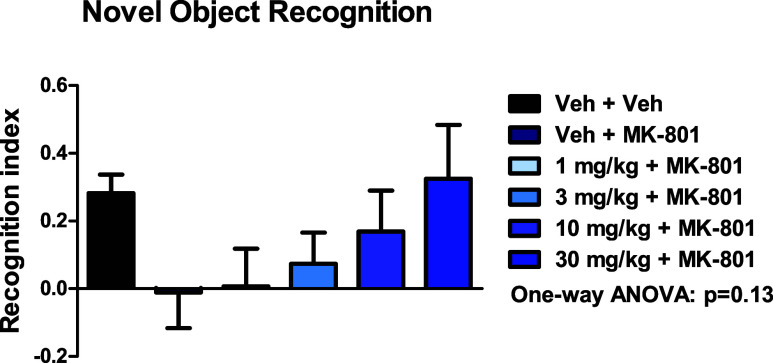
Rat MK-801-induced disruption of novel object recognition and reversal by VU6033685 (**8**). MK-801 (0.075 mg/kg SC) induced a robust disruption of NOR, which was dose-dependently reversed by oral administration (0.5% Natrasol/0.015% Tween 80 in water). *N* = 12–15/group of male Sprague–Dawley rats. One-way ANOVA: *p* = 0.13.

Based on our previous work with mGlu_1_ PAMs,^25^ we next evaluated **8** for its ability to reverse MK-801 disruptions of novel object recognition (NOR) in rats (Figure 7). While not as robust as PAM **3**, **8** did dose-dependently reverse the deficits induced by MK-801. Exposures with this vehicle (0.5% Natrasol/0.015% Tween 80 in water) at the 30 mg/kg dose achieved free brain levels ∼5.2-fold the rat *in vitro* EC_50_ (579 nM).^27^

### Dopamine Release

**Figure 8:**
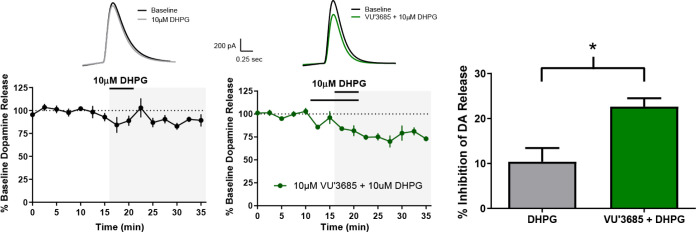
mGlu_1_-mediates DHPG induced Reductions in Striatal DA Release via PAM **8**. Effects of 10 μM DHPG, a group I mGlu receptor agonist, in the absence or presence of 10 μM PAM 8 on electrically evoked striatal DA release. All experiments were performed in the presence of nAChR antagonist (1 μM DHβE). *N* = 5–6 slices per condition (slices were made from 5 separate mice).

Previously, with PAMs **1** and **2**, we demonstrated that activation of mGlu_1_ reduces striatal DA release via activation of CB_2_ cannabinoid receptors.^11^ To confirm that the structurally distinct mGlu_1_ PAM **8** has a similar effect on dopamine (DA) release, we determined the effect of **8** on a subthreshold concentration of the Group I mGlu agonist DHPG (10 μM). Application of 10 μM DHPG alone did not produce a significant inhibition of striatal DA release. However, this subthreshold concentration of DHPG induced a robust inhibition of DA release (Figure 8) when coapplied with PAM **8** (10 μM). These results suggest that activation of mGlu_1_ inhibits stimulus-induced DA release in the striatum for multiple mGlu_1_ PAM chemotypes.^27^

### The Emergence of Adverse Events (AEs)

At this point, the project team was focused on derisking a novel mGlu_1_ PAM chemotype and assessing if the AEs observed with **3**^25^ would be noted with **8**. For many CNS targets, mice are more sensitive to overstimulation than rats. Thus, we performed a dose escalation PO PK study in mice (50, 150, and 500 mg/kg) with PAM **8** (mouse EC_50_ = 52 nM, 102%), and achieved >24-fold the EC_50_ free brain concentration (∼1250 nM) without any observed AEs. At the 500 mg/kg dose, ∼90-fold mouse free brain concentration was achieved, and all animals displayed uncoordinated movements and were cold to the touch. Thus, we initiated a three-day dose escalation toxicology study in mice at doses of 50, 200, and 400 mg/kg with six male mice per dose group to explore AEs above the anticipated human *C*_max_ of 4 μM and AUC_ss_ of 62 μM·h. At 50 mg/kg (AUC_ss_ of 5.5 μM·h, 0.09 multiple of AUC), there were no test-item-related findings. At 200 mg/kg (AUC_ss_ of 69.9 μM·h, 1.1 multiple of AUC), some of the mice showed signs of weight loss, decreased motor activity, and swaying gait. In the high-dose group (500 mg/kg, AUC_ss_ of 208 μM·h, 3.4 multiple of AUC), several mice displayed weight loss, decreased motor activity, and swaying gait. Overall, a maximum tolerated dose (MTD) from this study was not reached. Turning our attention back to the rat, we had noted in the NOR study that free brain concentrations up to 5.3-fold the rat EC_50_ were well tolerated. However, subsequent PO PK studies that reached 6.2-fold the rat EC_50_ free in brain (664 nM) resulted in piloerection, slow movement, and reaction to stimuli which persisted for ∼2 h postdose. This was surprising, as PAM **3** only exhibited AEs in dogs.^25^ To assess potential AEs in dogs, we performed a single, low-dose (0.5 mg/kg) IV bolus of PAM **8** to male beagle dogs. PAM **8** possessed a good PK profile in dogs (CL_p_ = 10 mL/min/kg, *t*_1/2_= 6.5 h, *V*_ss_ = 4.6 L/kg); however, all dogs displayed dizziness, salivation, and uncoordinated movements. Combined, these data halted further progression of VU6033685/BI1752 (**8**) and required the team to pause and postulate the origins of these AEs with mGlu_1_ PAMs.

## Conclusions

In summary, we disclose the further optimization of metabotropic glutamate receptor subtype 1 (mGlu_1_) positive allosteric modulator (PAM) VU6024578/BI02982816 (**3**) and the discovery of a chemically distinct mGlu_1_ PAM, VU6033685/BI1752 (**8**), to evaluate efficacy and tolerability. PAM **8** was potent, selective, CNS penetrant, and efficacious in both AHL and NOR. Importantly, the furanyl moiety (a potential toxicophore) of **3** was replaced by an *N*-linked pyrazole in **8**. Unlike PAM **3**, AEs with **8** were noted not only in dogs but also in mice and rats, which precluded further advancement. The mGlu_1_ PAM mechanism has strong human genetic support and robust efficacy in preclinical models of psychosis and cognition; however, the origins of the AEs are unclear. Are they target mediated? Like mGlu_5_ PAMs, is signal bias the key to avoiding AEs? Could the activation of an mGlu_1_/mGlu_5_ heterodimer be responsible for the AEs? The project team once again shifted resources to evaluate a third distinct chemotype while delving into a deeper mechanistic exploration of mGlu_1_ activation. Progress toward these possible origins of the AEs will be reported in due course.

## Supporting Information Available

Additional experimental details, methods for the synthesis and characterization of all compounds (^1^H and ^13^C NMR, HPLC), *in vitro* and *in vivo* DMPK protocols and supplemental figures (PDF)

## Abbreviations

PAM: positive allosteric modulator
PBL: plasma/brain level
DMPK: drug metabolism and pharmacokinetics
AE: adverse event
mGlu_1_: metabotropic glutamate receptor subtype 1
MED: minimum effective dose
NOR: novel object recognition
AHL: amphetamine-induced hyperlocomotion
