# Supplementary material for: Further Optimization of the mGlu1 PAM VU6024578/BI02982816: Discovery and Characterization of VU6033685
Source: ACS Chem Neurosci. 2025 Feb 5;16(4):745–52. doi: 10.1021/acschemneuro.5c00014 (PMC11843613; doi:10.1021/acschemneuro.5c00014)
Supplement: Supplementary file 1 — cn5c00014_si_001.pdf [file cn5c00014_si_001.pdf]

Further optimization of the mGlu$_1$ PAM VU6024578/BI02982816: Discovery and characterization of VU6033685

Carson W. Reed,[1,2] Jacob F. Kalbfleisch,[1,2] Jeremy A. Turkett,[1,2] Trevor A. Trombley,[1,2] Paul K. Spearing,[1,2] Daniel H. Haymer,[1,2] Marc Quitalig,[1,2] Jonathan W. Dickerson,[1,2] Daniel J. Foster,[1,2] Annie L. Blobaum, [1,2] Olivier Boutaud,[1,2] Hyekyung P. Cho,[1,2] Colleen M. Niswender,[1,2,4,5,6] Jerri M. Rook,[1,2] Henning Priepke,[7] Heiko Sommer,[7] Stefan Scheuerer,[7] Daniel Ursu,[7] P. Jeffrey Conn,[1,2,4,5,6] Bruce J. Melancon[1,2] and Craig W. Lindsley[1,2,3]*

Affiliation:
[1]Warren Center for Neuroscience Drug Discovery, Vanderbilt University, Nashville, TN 37232, USA
[2]Department of Pharmacology, Vanderbilt University School of Medicine, Nashville, TN 37232, USA
[3]Department of Chemistry, Vanderbilt University, Nashville TN 37232, USA
[4]Vanderbilt Kennedy Center, Vanderbilt University Medical Center, Nashville, TN 37232, USA
[5]Vanderbilt Brain Institute, Vanderbilt University, Nashville, TN 37232, USA
[6]Vanderbilt Institute of Chemical Biology, Vanderbilt University, Nashville, TN 37232, USA
[7]Boehringer Ingelheim Pharma GmbH & Co. KG, Birkendorfer Str. 65 | 88397 Biberach, Germany
*To whom correspondence should be addressed at craig.lindsley@vanderbilt.edu

Table of Contents

General synthetic methods and instrumentation……………………………………………..S2

Synthesis of VU6026104 (**4**)……………………………………………………………S4

Synthesis of benzoic acid intermediate (**12**)…………………………………………….S5

Synthesis of VU6026095 (**5**)……………………………………………………………S7

Synthesis of VU6028266 (**6**)…………………………………………………………....S8

Synthesis of VU6030257 (**7**)……………………………………………………………S9

Synthesis of VU6033685 (**8**)…………………………………………………………..S10

In Vitro Molecular Pharmacology Methods……………………………………………S12

In vitro and in vivo DMPK Methods……………………………………………………S13

Behavioral Pharmacology Methods……………………………………………………..S17

Brain Slice Preparation and Fast Scanning Cyclic Voltammetry……………………..…S18

General synthetic methods and instrumentation

All NMR spectra were recorded on a 400 MHz AMX Bruker NMR spectrometer. ^{1}H and ^{13}C chemical shifts are reported in δ values in ppm downfield with the deuterated solvent as the internal standard. Data are reported as follows: chemical shift, multiplicity (s = singlet, d = doublet, t = triplet, q = quartet, b = broad, m = multiplet), integration, coupling constant (Hz). Low resolution mass spectra were obtained on an Agilent 6120 or 6150 with ESI source. MS parameters were as follows: fragmentor: 70, capillary voltage: 3000 V, nebulizer pressure: 30 psig, drying gas flow: 13 L/min, drying gas temperature: 350 ºC. Samples were introduced via an Agilent 1290 UHPLC comprised of a G4220A binary pump, G4226A ALS, G1316C TCC, and G4212A DAD with ULD flow cell. UV absorption was generally observed at 215 nm and 254 nm with a 4 nm bandwidth. Column: Waters Acquity BEH C18, 1.0 x 50 mm, 1.7 um. Gradient conditions: 5% to 95% CH_3CN in H_2O (0.1% TFA) over 1.4 min, hold at 95% CH_3CN for 0.1 min, 0.5 mL/min, 55 ºC. High resolution mass spectra were obtained on an Agilent 6540 UHD Q-TOF with ESI source. MS parameters were as follows: fragmentor: 150, capillary voltage: 3500 V, nebulizer pressure: 60 psig, drying gas flow: 13 L/min, drying gas temperature: 275 ºC. Samples were introduced via an Agilent 1200 UHPLC comprised of a G4220A binary pump, G4226A ALS, G1316C TCC, and G4212A DAD with ULD flow cell. UV absorption was observed at 215 nm and 254 nm with a 4 nm bandwidth. Column: Agilent Zorbax Extend C18, 1.8 µm, 2.1 x 50 mm. Gradient conditions: 5% to 95% CH_3CN in H_2O (0.1% formic acid) over 1 min, hold at 95% CH_3CN for 0.1 min, 0.5 mL/min, 40 ºC. For compounds that were purified on a Gilson preparative reversed-phase HPLC, the system comprised of a 333 aqueous pump with solvent-selection valve, 334 organic pump, GX-271 or GX-281 liquid hander, two column switching valves, and a 155 UV detector. UV

wavelength for fraction collection was user-defined, with absorbance at 254 nm always monitored. Method 1: Phenomenex Axia-packed Luna C18, 30 x 50 mm, 5 µm column. Mobile phase: CH_3CN in H_2O (0.1% TFA). Gradient conditions: 0.75 min equilibration, followed by user defined gradient (starting organic percentage, ending organic percentage, duration), hold at 95% CH_3CN in H_2O (0.1% TFA) for 1 min, 50 mL/min, 23 ºC. Method 2: Phenomenex Axia-packed Gemini C18, 50 x 250 mm, 10 um column. Mobile phase: CH_3CN in H_2O (0.1% TFA). Gradient conditions: 7 min equilibration, followed by user defined gradient (starting organic percentage, ending organic percentage, duration), hold at 95% CH_3CN in H_2O (0.1% TFA) for 7 min, 120 mL/min, 23 ºC. All commercial reagents were used as received without further purification. All final compounds were purified to >95% as determined by analytical LCMS (214 nm, 254 nm, and ELSD), ^{1}H and/or ^{13}C NMR, and high-resolution MS.

Synthesis of 2-chloro-*N*-((1,1'-dimethyl-1*H*,1'*H*-[3,4'-bipyrazol]-5-yl)methyl)-5-(furan-2-yl)benzamide (VU6026104) (4)

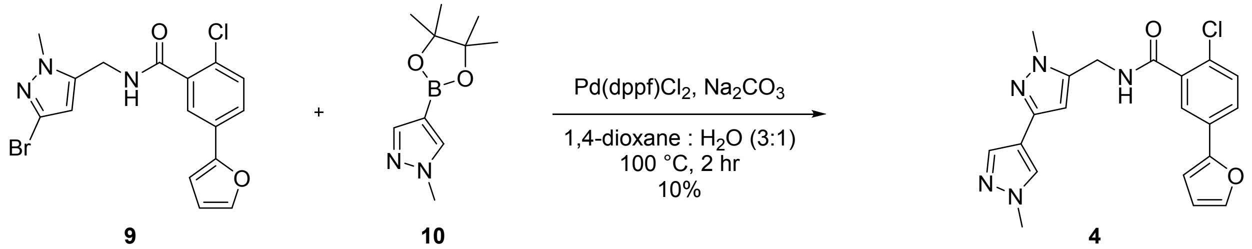

A vial was sequentially charged with *N*-[(5-bromo-2-methyl-pyrazol-3-yl)methyl]-2-chloro-5-(2-furyl)benzamide (**9**) (42 mg, 0.1 mmol), 1-methylpyrazole-4-boronic acid pinacol ester (**10**) (33 mg, 0.2 mmol), sodium carbonate (35 mg, 0.3 mmol), Pd(dppf)Cl_2 (7.8 mg, 0.01 mmol), 1,4-dioxane (0.4 mL), and H_2O (0.1 mL). The reaction was degassed then flushed w/ N_2 (g) (x2) before being heated to 90 °C for 90 min. Upon cooling to rt, the crude reaction mixture was diluted with EtOAc, filtered, concentrated, dissolved in DMSO (1 mL), and purified via reverse phase HPLC (Gilson 30x100mm basic C18 column: H_2O with 0.05% NH_4OH / MeCN: 5-95% MeCN gradient) to afford the desired product (**4**) (4.2 mg, 0.011 mmol, 10 % yield) as a white solid.

^{1}H NMR (400 MHz, DMSO-d$_6$): 9.07 (t, *J* = 5.6 Hz, 1H), 7.95 (s, 1H), 7.80 - 7.74 (m, 3H), 7.66 (s, 1H), 7.56 (d, *J* = 8.1 Hz, 1H), 7.12 (d, *J* = 3.3 Hz, 1H), 6.63 (dd, *J* = 3.4, 1.8 Hz, 1H), 6.37 (s, 1H), 4.52 (d, *J* = 4.9 Hz, 2H), 3.84 (s, 3H), 3.82 (s, 3H).

^{13}C NMR (100 MHz, DMSO-d$_6$): 166.4, 151.6, 144.2, 143.1, 140.8, 137.4, 136.4, 130.9, 129.7, 128.9, 128.1, 125.7, 124.1, 116.4, 113.1, 107.9, 103.3, 60.3, 36.7, 34.2.

HRMS: (Q-TOF, ES+): calc'd for $C_{20}H_{19}ClN_5O_2$ $(M+H)^+$, 396.1222; found, 396.1224.

Synthesis of 2-chloro-5-(1*H*-pyrazol-1-yl)benzoic acid (12)

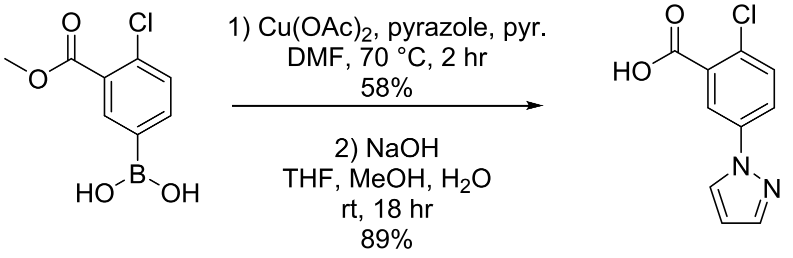

11 **12**

Step 1

A RBF was charged with 4-chloro-3-(methoxycarbonyl)phenylboronic acid (25 g, 117 mmol), pyrazole (15.9 g, 233 mmol), copper (II) acetate (42.4 g, 233 mmol), DMF (200 mL), and pyridine (18.9 mL, 233 mmol). The reaction was then vigorously stirred at 70 ^{o}C for 4 hr. The solution was filtered through celite, then diluted with EtOAc, washed with H_2O (3x) and brine, dried over Na_2SO_4 then concentrated. The residue was purified by flash chromatography (Teledyne ISCO flash purification system; silica gel column; hexanes : EtOAc; 0-25% EtOAc gradient) to afford the intermediate methyl ester (7.9 g, 33.4 mmol, 29% yield) as a white solid.

^{1}H NMR (400 MHz, DMSO-d_6): 8.61 (d, *J* = 2.8 Hz, 1H), 8.28 (d, *J* = 2.8 Hz, 1H), 8.06 (dd, *J* = 8.8, 2.8 Hz, 1H), 7.80 (d, *J* = 1.6 Hz, 1H), 7.71 (d, *J* = 8.8 Hz, 1H), 6.59 (dd, *J* = 2.6, 1.8 Hz, 1H), 3.90 (s, 3H).

^{13}C NMR (100 MHz, DMSO-d_6): 164.9, 141.7, 138.4, 131.9, 131.0, 128.6, 128.2, 122.2, 120.2, 108.6, 52.8.

HRMS: (Q-TOF, ES+): calc'd for $C_{11}H_{10}ClN_2O_2$ $(M+H)^+$, 237.0425; found, 237.0425.

Step 2

The intermediate methyl ester from above (7.9 g, 33.4 mmol) and sodium hydroxide (4.11 g, 100 mmol) in THF (100 mL), H_2O (15 mL) and MeOH (5 mL) was stirred at rt for 18 hr. The solution was concentrated, and the resulting residue was taken up in H_2O (10 mL) and saturated with NaCl. The pH was adjusted to ~6.0 with HCl (1.0 N aq.) The resulting solids were collected via vacuum filtration, washing with a small amount of H_2O, and then dried to give 1.27 g of desired product. The filtrate was then saturated with NaCl at which point additional product crashed out. The suspended solids were decanted from the excess solid NaCl, collected by vacuum filtration and dried. The solids were then dissolved in MeOH (sonicated until a small amount of solid remained insoluble.) The suspension was filtered, and the filtrate was concentrated to afford an additional 5.44 g of desired product. All crops were combined to afford the desired product (**12**) (6.71 g, 30.1 mmol, 90% yield) as a white solid.

^{1}H NMR (400 MHz, DMSO-d_6): 8.61 (d, *J* = 2.6 Hz, 1H), 8.20 (d, *J* = 2.8 Hz, 1H), 7.98 (dd, *J* = 8.8, 2.8 Hz, 1H), 7.79 (d, *J* = 1.7 Hz, 1H), 7.65 (d, *J* = 8.7 Hz, 1H), 6.59 – 6.56 (bm, 1H)

^{13}C NMR (100 MHz, DMSO-d_6): 166.3, 141.6, 138.3, 133.4, 131.6, 128.3, 128.2, 121.4, 119.8, 108.5

HRMS: (Q-TOF, ES+): calc'd for $C_{10}H_8ClN_2O_2$ $(M+H)^+$, 223.0269; found, 223.0269.

Synthesis of 2-chloro-*N*-((1,2'-dimethyl-1*H*,2'*H*-[3,3'-bipyrazol]-5-yl)methyl)-5-(1*H*-pyrazol-1-yl)benzamide (VU6026095) (5)

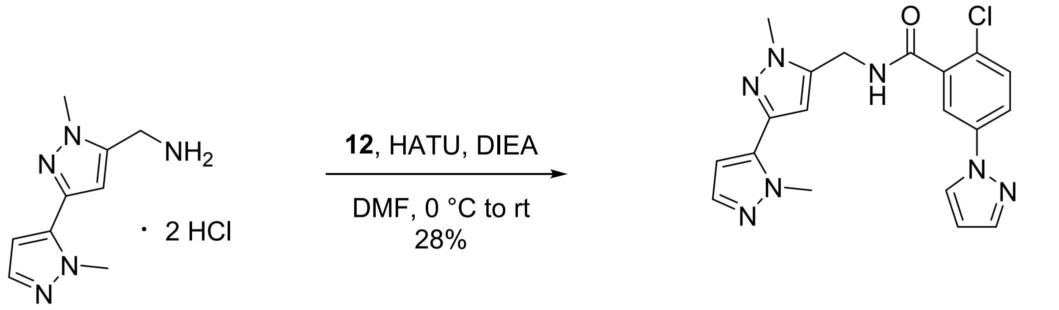

13 **5**

A suspension of 2-methyl-5-(2-methylpyrazol-3-yl)pyrazol-3-yl]methanamine dihydrochloride (**13**) (220 mg, 0.8 mmol), benzoic acid (**12**) (185 mg, 0.8 mmol) and *N*,*N*-diisopropylethylamine (0.6 mL, 3.3 mmol) in DMF (2.2 mL) was sonicated until everything was in solution. The resulting solution was then cooled to 0 °C. HATU (474 mg, 1.3 mmol) was added in portions, and then the solution was stirred at rt for 2 hr. The solution was diluted with EtOAc, washed with H_2O (2x) and brine, dried over Na_2SO4 then concentrated. The residue was purified by flash chromatography (Teledyne ISCO flash purification system; silica gel column; DCM : acetone; 0-40% acetone gradient) to afford the desired product (**5**) (92 mg, 0.23 mmol, 28% yield) as a white solid.

^1H NMR (400 MHz, DMSO-d_6): 9.15 (t, *J* = 5.6 Hz, 1H), 8.61 (d, *J* = 2.6 Hz, 1H), 7.96 – 7.93 (m, 2H), 7.79 (d, *J* = 1.8 Hz, 1H), 7.66 – 7.62 (m, 1H), 7.40 (d, *J* = 1.9 Hz, 1H), 6.61 – 6.57 (m, 2H), 6.49 (d, *J* = 1.9 Hz, 1H), 4.59 (d, *J* = 5.6 Hz, 2H), 4.04 (s, 3H), 3.92 (s, 3H)

^{13}C NMR (100 MHz, DMSO-d_6): 165.6, 141.6, 140.7, 140.4, 138.3, 137.8, 137.3, 135.9, 130.8, 128.2, 126.9, 120.3, 118.3, 108.4, 105.2, 104.9, 38.5, 36.7, 33.8

HRMS: (Q-TOF, ES+): calc'd for $C_{19}H_{19}ClN_7O$ $(M+H)^+$, 396.1334; found, 396.1333.

Synthesis of 2-chloro-*N*-((2-methylquinolin-3-yl)methyl)-5-(1*H*-pyrazol-1-yl)benzamide (VU6028266) (6)

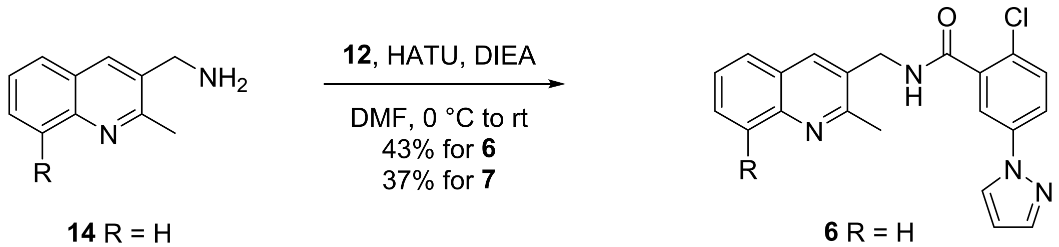

14 R = H
15 R = F

6 R = H
7 R = F

Benzoic acid (**12**) (259 mg, 1.2 mmol) was combined with HATU (530 mg, 1.4 mmol) and *N*,*N*-diisopropylethylamine (1.0 mL, 5.8 mmol) in DMF (5.8 mL) and stirred at 0 °C for 15 min. (2-methyl-3-quinolyl)methanamine (**14**) (200 mg, 1.2 mmol) was then added, and the reaction was allowed to warm to r.t. and stirred for 30 min. The reaction was quenched with the addition of 1N aq. NaOH (15 mL) and extracted with 3:1 $CHCl_3$/IPA (20 mL x 3). The combined organic phase was then passed through a phase separator before being concentrated and purified via flash chromatography (Teledyne ISCO flash purification system; silica gel column; hexanes : EtOAc; 10-60% EtOAc gradient) to afford the desired product (**6**) (186 mg, 0.49 mmol, 43% yield) as a white powder.

^{1}H NMR (400 MHz, DMSO-d_6): 9.18 (t, *J* = 5.6 Hz, 1H), 8.63 (d, *J* = 2.5 Hz, 1H), 8.22 (s, 1H), 8.01 (d, *J* = 2.6 Hz, 1H), 7.97 – 7.90 (m, 3H), 7.80 (s, 1H), 7.72 – 7.63 (m, 2H), 7.54 (t, *J* = 7.5 Hz, 1H), 6.60 – 6.58 (m, 1H), 4.66 (d, *J* = 5.6 Hz, 2H), 2.73 (s, 3H)

^{13}C NMR (100 MHz, DMSO-d_6): 165.8, 157.8, 146.3, 141.6, 138.3, 137.6, 134.1, 130.8, 130.4, 129.1, 128.2, 127.9, 127.5, 126.9, 126.6, 125.9, 120.2, 118.3, 108.4, 40.7, 22.8

HRMS: (Q-TOF, ES+): calc'd for $C_{21}H_{18}ClN_4O$ $(M+H)^+$, 377.1164; found, 377.1163.

Synthesis of 2-chloro-*N*-((8-fluoro-2-methylquinolin-3-yl)methyl)-5-(1*H*-pyrazol-1-yl)benzamide (VU6030257) (7)

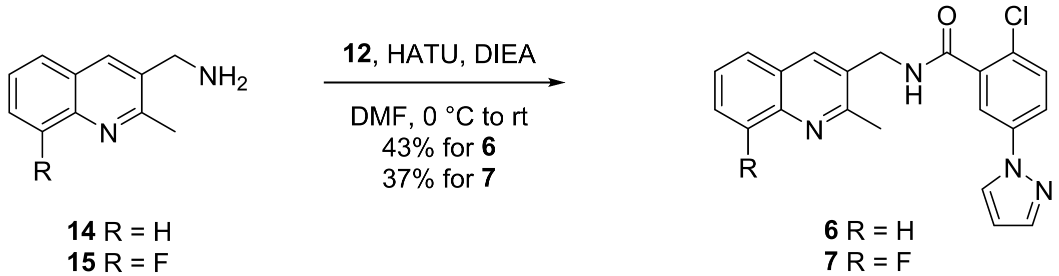

Benzoic acid (**12**) (310 mg, 1.4 mmol) was combined with HATU (635 mg, 1.7mmol) and *N*,*N*-diisopropylethylamine (1.2 mL, 7.0 mmol) in DMF (5 mL) and cooled to 0 °C. The reaction was stirred at this temperature for 15 min before the addition of (8-fluoro-2-methyl-3-quinolyl)methanamine;dihydrochloride (**15**) (366 mg, 1.4 mmol). The reaction was allowed to warm to r.t. and stirred for 30 min. The reaction was quenched with the addition of 1N aq. NaOH (1.5 mL) with vigorous stirring. Additional water was added, and the resulting solution was extracted with 3:1 $CHCl_3$/IPA (3 mL x 3). The combined organics were passed through a phase separator, concentrated, and purified via flash chromatography (Teledyne ISCO flash purification system; silica gel column; hexanes : EtOAc; 10-60% EtOAc gradient). Fractions containing the desired product were concentrated under reduced pressure before being redissolved in the minimum volume of 3:1 EtOH/H_2O and recrystallized over 48 hr. The resulting white crystals were isolated via vacuum filtration to afford the desired product (**7**) (203 mg, 0.51 mmol, 37% yield).

^{1}H NMR (400 MHz, DMSO-d_6): 9.20 (t, *J* = 5.7 Hz, 1H), 8.63 (d, *J* = 2.6 Hz, 1H), 8.28 (s, 1H), 8.02 (d, *J* = 2.7 Hz, 1H), 7.96 (dd, *J* = 8.8, 2.7 Hz, 1H), 7.81 – 7.73 (m, 2H), 7.66 (d, *J* = 8.7 Hz, 1H), 7.55 – 7.48 (m, 2H), 6.59 (t, *J* = 2.2 Hz, 1H), 4.68 (d, *J* = 5.6 Hz, 2H), 2.76 (s, 3H)

^{13}C NMR (100 MHz, DMSO-d_6): 165.9, 158.4, 156.9 (d, J_{CF} = 252.4 Hz), 141.6, 138.3, 137.5, 136.0 (d, J_{CF} = 11.3 Hz), 133.9 (d, J_{CF} = 2.6 Hz), 131.7, 130.8, 128.4 (d, J_{CF} = 2.3

Hz), 128.2, 126.9, 125.9 (d, J_{CF} = 8.0 Hz), 123.4 (d, J_{CF} = 4.4 Hz), 120.3, 118.3, 113.3 (d, J_{CF} = 18.4 Hz), 108.4, 40.6, 22.9

HRMS: (Q-TOF, ES+): calc'd for $C_{21}H_{17}ClFN_4O$ $(M+H)^+$, 395.1069; found, 395.1072.

Synthesis of 2-chloro-*N*-((6-cyclopropyl-2-methylpyridin-3-yl)methyl)-5-(1*H*-pyrazol-1-yl)benzamide (VU6033685) (8)

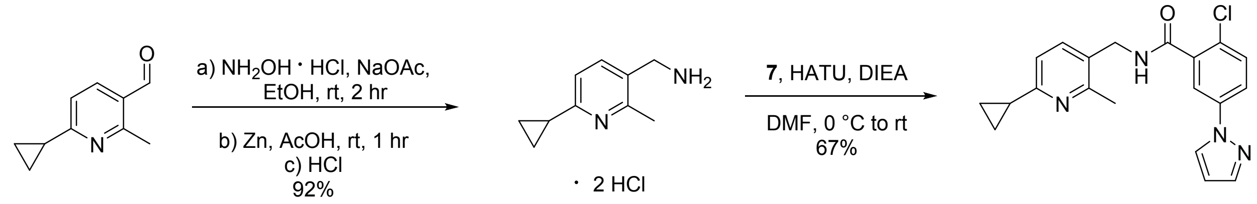

16 **17** **8**

Step 1

A suspension of 6-cyclopropyl-2-methyl-pyridine-3-carbaldehyde (**16**) (1.7 g, 10.6 mmol), hydroxylamine hydrochloride (774 mg, 11.1 mmol), and sodium acetate (925 mg, 11.1 mmol) in EtOH (30 mL) was stirred at rt for 2 hr. The solution was diluted with EtOAc, washed with H_2O and brine, dried over Na_2SO_4, and then concentrated.To the resulting residue was added zinc powder (3.5 g, 53 mmol) and AcOH (15 mL). The mixture was stirred at rt for an additional 1 hr. The mixture was filtered, and the filtrate was concentrated. The residue was dissolved in THF (25 mL) then added slowly to HCl (50 mL, 4 M solution in 1,4-dioxane) with vigorous stirring. The resulting solids were collected via vacuum filtration (washing with THF) and dried under vacuum to afford intermediate **17** (2.3 g, 9.74 mmol, 92% yield) as an off-white solid.

Note: **17** was observed to decompose upon storage. As a result, **17** was carried forward as is to the next step.

HRMS: (Q-TOF, ES+): calc'd for $C_{10}H_{15}N_2$ $(M+H)^+$, 163.1230; found, 163.1227.

Step 2

HATU (2.3 g, 6.0 mmol) was added to a solution of (6-cyclopropyl-2-methyl-3-pyridyl)methanamine;dihydrochloride (**17**) (1.2 g, 5.0 mmol), benzoic acid (**12**) (1.1 g, 5.0 mmol) and *N*,*N*-diisopropylethylamine (3.0 mL, 17.4 mmol) in DMF (20 mL) cooled

to 0 ˚C portionwise. Upon completion of addition, the reaction was warmed to rt and stirred for 2 hr. The solution was then added slowly to H_2O (300 mL) with vigorous stirring. The resulting solids were collected via vacuum filtration (washing with H_2O) and dried under vacuum. The solid was then dissolved in 1:1 EtOAc/MeOH (~500 mL) and filtered through a pad of Celite to remove a fine insoluble material. The solution was then concentrated, and the solids were suspended in toluene (100 mL) and sonicated for 10 min. The resulting mixture was filtered, and the solid was washed with toluene and then dried under vacuum to afford the desired product (**8**) (1.2 g, 3.33 mmol, 67% yield) as a white solid.

^{1}H NMR (400 MHz, DMSO-d_6): 8.99 (t, *J* = 5.7 Hz, 1H), 8.61 (d, *J* = 2.6 Hz, 1H), 7.94 – 7.91 (m, 2H), 7.79 (d, *J* = 1.8 Hz, 1H), 7.64 – 7.61 (m, 1H), 7.54 (d, *J* = 7.8 Hz, 1H), 7.08 (d, *J* = 7.8 Hz, 1H), 6.58 (t, *J* = 2.1 Hz, 1H), 4.41 (d, *J* = 5.6 Hz, 2H), 2.45 (s, 3H), 2.05 – 1.99 (m, 1H), 0.91 – 0.86 (m, 4H).

^{13}C NMR (100 MHz, DMSO-d_6): 165.6, 159.7, 155.4, 141.6, 138.3, 137.6, 135.9, 130.8, 128.24, 128.15, 126.9, 120.1, 118.5, 118.3, 108.4, 40.1, 22.1, 16.3, 9.3

HRMS: (Q-TOF, ES+): calc'd for $C_{20}H_{20}ClN_4O$ $(M+H)^+$, 367.1320; found, 367.1323.

In vitro Pharmacology

Molecular Pharmacology. Tetracycline-tested fetal bovine serum (FBS) was purchased from Atlanta Biologicals (Lawrenceville, GA), and all other tissue culture reagents and Fluo-4-acetoxymethylester (Fluo-4-AM) were purchased from Life Technologies (Carlsbad, CA). Tetracycline hydrochloride (Sigma), L-glutamic acid (Tocris, Minneapolis, MN), and (*S*)-3,5-dihydroxyphenylglycine (DHPG) (Abcam, Cambridge, MA). EZ-Link Sulfo-NHS-SS-Biotin and NeutrAvidin agarose beads (Pierce Biotechnology, Rockford, IL).

Cell Culture and Mutagenesis. Tetracycline-inducible human $mGlu_1$ WT-T-REx^{TM}-293 cells (Wu et al, Science 2014) were cultured at 37 ºC in Dulbecco's Modified Eagle Medium (DMEM) growth medium containing 10% Tet-tested FBS, 2 mM L-glutamine, 20 mM HEPES, 0.1 mM non-essential amino acids, 1 mM sodium pyruvate, antibiotic/antimycotic, 100 µg/mL hygromycin and 5 µg/mL blasticidin in the presence of 5% CO_2. To generate a collection of stable cell lines carrying tetracycline-inducible $hmGlu_1$ schizophrenic mutants,[21,22] site-directed mutagenesis of human $mGlu_1$ WT in pcDNA5/TO was performed using Quikchange II XL kit (Agilent Technologies, Santa Clara, CA), and all point-mutations were confirmed by sequencing. The mutant stable cell lines were generated in the same manner as the WT as previously described and cultured in the growth medium described above.

Calcium Mobilization Assay. To determine the potency of $mGlu_1$ PAMs in calcium assays, Ca flux was measured as previously described in *ACS Chem. Bio*. **2014**, *9*, 2334-2346. Briefly, the day before the assay, human $mGlu_1$ WT-T-REx^{TM}-293 cells were plated in black-walled, clear-bottomed, poly-D-lysine coated 96-well plates at 80,000 cells/100 µL assay medium (DMEM supplemented with 10% dialyzed FBS, 20 mM HEPES, and 1 mM sodium pyruvate) containing 50 ng/mL of tetracycline to induce $mGlu_1$ expression. The next day, media was removed and the cells were incubated with 50 µL of 1.15 µM Fluo-4 AM dye solution prepared in assay buffer (buffer (Hank's balanced salt solution, 20 mM HEPES, and 2.5 mM probenecid) for 45 min at 37 °C, the dye was removed and replaced with 45 µL of assay buffer. Then, calcium flux was measured using Flexstation II (Molecular Devices, Sunnyvale, CA). Compounds serially diluted

at half log concentrations in DMSO were further diluted in assay buffer. The compounds or DMSO vehicle were added to cells and incubated for 2.5 min and an EC_{20} concentration of glutamate was added and incubated for 1 min. An EC_{max} concentration of glutamate was also added to cells that were incubated with DMSO vehicle to accurately calculate the EC_{20} calcium response. Data were normalized by subtracting the basal florescent peak before EC_{20} agonist addition from the maximal peak elicited by EC_{20} agonist and PAMs. Using GraphPad Prism 5.0, the concentration response curves were generated and the potencies of the $mGlu_1$ PAMs were determined.

DMPK Methods

Plasma protein binding: The protein binding of each compound was determined in plasma via equilibrium dialysis employing RED Plates (ThermoFisher Scientific, Rochester, NY). Plasma was added to the 96 well plate containing test compound and mixed thoroughly for a final concentration of 5 μM. Subsequently, an aliquot of the plasma-compound mixture was transferred to the *cis* chamber (red) of the RED plate, with an phosphate buffer (25 mM, pH 7.4) in the *trans* chamber. The RED plate was sealed and incubated for 4 hours at 37°C with shaking. At completion, aliquots from each chamber were diluted 1:1 with either plasma (*cis*) or buffer (*trans*) and transferred to a new 96 well plate, at which time ice-cold acetonitrile containing internal standard (50 ng/mL carbamazepine) (2 volumes) was added to extract the matrices. The plate was centrifuged (3000 rcf, 10 min) and supernatants transferred and diluted 1:1 (supernatant: water) into a new 96 well plate, which was then sealed in preparation for LC/MS/MS analysis. Each compound was assayed in triplicate within the same 96-well plate. Fraction unbound was determined using the following equation

$$F_u = \frac{Conc_{buffer}}{Conc_{plasma}}$$

Intrinsic clearance: Human or rat hepatic microsomes (0.5 mg/mL) and 1 μM test compound were incubated in 100 mM potassium phosphate pH 7.4 buffer with 3 mM $MgCl_2$ at 37°C with constant shaking. After a 5 min preincubation, the reaction was initiated by addition of NADPH (1 mM). At selected time intervals (0, 3, 7, 15, 25, and 45 min), aliquots were taken and subsequently placed into a 96-well plate containing cold acetonitrile with internal standard (50 ng/mL carbamazepine). Plates were then centrifuged at 3000 rcf (4° C) for 10 min, and the supernatant was transferred to a separate 96-well plate and diluted 1:1 with water for LC/MS/MS analysis. The *in vitro* half-life ($T_{1/2}$, min, Eq. 1), intrinsic clearance (CL_{int}, mL/min/kg, Eq. 2) and subsequent predicted hepatic clearance (CL_{hep}, mL/min/kg, Eq. 3) was determined

employing the following equations:

(1) $$T_{1/2} = \frac{Ln(2)}{k}$$

where k represents the slope from linear regression analysis of the natural log percent remaining of test compound as a function of incubation time

(2) $$CL_{\text{int}} = \frac{0.693}{in\,vitro\,T_{1/2}}\,x\,\frac{mL\ incubation}{mg\ microsomes}\,x\,\frac{45\,mg\,microsomes}{gram\ liver}\,x\,\frac{20^{a}\ gram\ liver}{kg\ body\ wt}$$

[a]scale-up factors: of 20 (human) or 45 (rat)

(3) $$CL_{hep} = \frac{Q_h \cdot CL\text{int}}{Q_h + CL\text{int}}$$

where Q_h (hepatic blood flow, mL/min/kg) is 21 (human) or 70 (rat).

LC/MS/MS Bioanalysis of Samples from Plasma Protein Binding and Intrinsic Clearance Assays. Samples were analyzed on a Thermo Electron TSQ Quantum Ultra triple quad mass spectrometer (San Jose, CA) via electrospray ionization (ESI) with two Themo Electron Accella pumps (San Jose, CA), and a Leap Technologies CTC PAL autosampler (Carrboro, NC). Analytes were separated by gradient elution on a dual column system with two Thermo Hypersil Gold (2.1 x 30 mm, 1.9 µm) columns (San Jose, CA) thermostated at 40°C. HPLC mobile phase A was 0.1% formic acid in water and mobile phase B was 0.1% formic acid in acetonitrile. The gradient started at 10% B after a 0.2 min hold and was linearly increased to 95% B over 0.8 min; hold at 95% B for 0.2 min; returned to 10% B in 0.1 min. The total run time was 1.3 min and the HPLC flow rate was 0.8 mL/min. While pump 1 ran the gradient method, pump 2 equilibrated the alternate column isocratically at 10% B. Compound optimization, data collection and processing was performed using Thermo Electron's QuickQuan software (v2.3) and Xcalibur (v2.0.7 SP1).

Inhibition of Cytochrome P450 Enzymes: A cocktail of substrates for cytochrome P450 enzymes (1A2: Phenacetin, 10 µM; 2C9: Diclofenac, 5 µM; 2D6: Dextromethorphan, 5 µM; 3A4: Midazolam, 2 µM) were mixed for cocktail analysis. For P450 2C19, the substrate stock (Mephenytoin, 40 µM)and substrate mix were prepared separately for discrete analysis. The

positive control for pan-P450 inhibition (miconazole) was included alongside each test compound in analysis.

A reaction mixture of 100 mM Kpi, pH 7.4, 0.1 mg/mL human liver microsomes (HLM) and Substrate Mix is prepared and aliquoted into a 96-deepwell block. Test compound and positive control (in duplicate) were then added such that the final concentration of test compound ranged from 0.1 – 30 μM. The plate was vortexed briefly and then pre-incubated at 37°C while shaking for 15 minutes. The reaction was initiated with the addition of NADPH (1 mM final concentration). The incubation continued for 8 min and the reaction quenched by 2x volume of cold acetonitrile containing internal standard (50 nM carbamazepine). The plate was centrifuged for 10 minutes (4000 rcf, 4°C) and the resulting supernatant diluted 1:1 with water for LC/MS/MS analysis. A 12 point standard curve of substrate metabolites over the range of 0.98 nM to 2000 nM.

Samples were analyzed via electrospray ionization (ESI) on an AB Sciex API-4000 (Foster City, CA) triple-quadrupole instrument that was coupled with Shimadzu LC-10AD pumps (Columbia, MD) and a Leap Technologies CTC PAL auto-sampler (Carrboro, NC). Analytes were separated by gradient elution using a Fortis C18 3.0 x 50 mm, 3 μm column (Fortis Technologies Ltd, Cheshire, UK) thermostated at 40°C. HPLC mobile phase A was 0.1% formic acid in water (pH unadjusted), mobile phase B was 0.1% formic acid in acetonitrile (pH unadjusted). The gradient started at 10% B after a 0.2 min hold and was linearly increased to 90% B over 1.2 min; held at 90% B for 0.1 min and returned to 10% B in 0.1 min followed by a re-equilibration (0.9 min). The total run time was 2.5 min and the HPLC flow rate was 0.5 mL/min. The source temperature was set at 500°C and mass spectral analyses were performed using multiple reaction monitoring (MRM), with transitions specific for each compound utilizing a Turbo-Ionspray® source in positive ionization mode (5.0 kV spray voltage).

The IC_{50} values for each compound were obtained for the individual CYP enzymes by quantitating the inhibition of metabolite formation for each probe substrate. A 0 μM compound condition (or control) was set to 100% enzymatic activity and the effect of increasing test compound concentrations on enzymatic activity could then be calculated from the % of control activity. Curves were fitted using XLfit 5.2.2 (four-parameter logistic model, equation 201) to determine the concentration that produces half-maximal inhibition (IC_{50}).

***In vivo* DMPK experimental**

Compounds were formulated as 10% tween 80 micro suspensions in sterile water at the concentration of 1 mg/ml and administered intraperitoneally to male Sprague- Dawley rats weighing 225 to 250 g (Harlan, Inc., Indianapolis, IN) at the dose of 10 mg/kg. The rat blood and brain were collected at 0.25 hr. Animals were euthanized and decapitated, and the brains were removed, thoroughly washed in cold phosphate buffered saline and immediately frozen on dry ice. Trunk blood was collected in EDTA Vacutainer tubes, and plasma was separated by centrifugation and stored at -80°C until analysis. Plasma was separated by centrifugation (4000 rcf, 4°C) and stored at 80°C until analysis. On the day of analysis, frozen whole-rat brains were weighed and diluted with 1:3 (w/w) parts of 70:30 isopropanol:water. The mixture was then subjected to mechanical homogenation employing a Mini-Beadbeater™ and 1.0 mm Zirconia/Silica Beads (BioSpec Products) followed by centrifugation. The sample extraction of plasma (20 μL) or brain homogenate (20 μL) was performed by a method based on protein precipitation using three volumes of ice-cold acetonitrile containing an internal standard (50 ng/mL carbamazepine). The samples were centrifuged (3000 rcf, 5 min) and supernatants transferred and diluted 1:1 (supernatant: water) into a new 96 well plate, which was then sealed in preparation for LC/MS/MS analysis.

In vivo samples were analyzed via electrospray ionization (ESI) on an AB Sciex API-5500 QTrap (Foster City, CA) instrument that was coupled with Shimadzu LC-20AD pumps (Columbia, MD) and a Leap Technologies CTC PAL auto-sampler (Carrboro, NC). Analytes were separated by gradient elution using a Fortis C18 3.0 x 50 mm, 3 μm column (Fortis Technologies Ltd, Cheshire, UK) thermostated at 40°C. HPLC mobile phase A was 0.1% formic acid in water (pH unadjusted), mobile phase B was 0.1% formic acid in acetonitrile (pH unadjusted). The gradient started at 30% B after a 0.2 min hold and was linearly increased to 90% B over 0.8 min; held at 90% B for 0.5 min and returned to 30% B in 0.1 min followed by a re-equilibration (0.9 min). The total run time was 2.5 min and the HPLC flow rate was 0.5 mL/min. The source temperature was set at 500°C and mass spectral analyses were performed using multiple reaction monitoring (MRM), with transitions specific for each compound utilizing a Turbo-Ionspray® source in positive ionization

mode (5.0 kV spray voltage). The calibration curves were constructed in blank plasma. All data were analyzed using AB Sciex Analyst software v1.5.1.

Animal care and use

All animal study procedures were approved by the Institutional Animal Care and Use Committee and were conducted in accordance with the National Institutes of Health regulations of animal care covered in Principles of Laboratory Animal Care (National Institutes of Health).

Data Analysis

The data for the studies were analyzed by a between-group 2 way analysis of variance for compound dose and time. Each dose group was compared with the vehicle control group. Total ambulation rotorod and catalepsy data was analyzed by one-way ANOVA. Each dose group was compared with the vehicle control group. The calculations were performed and graphed using GraphPad Prism (version 4.03, GraphPad, La Jolla, CA).

Amphetamine-induced hyperlocomotion (rat)

Adult male Sprague-Dawley rats placed into the activity chambers (Med Associates). Thirty minutes after placement in the chambers, the rats were administered VU6024578 (1-10 mg/kg p.o..) or vehicle. Sixty minutes later the rats were injected with amphetamine (0.75 mg/kg s.c. 1 ml/kg) or vehicle (saline) and placed back in the activity chambers for 90 minutes. The data for the studies were analyzed by a between-group 2 way analysis of variance for compound dose and time. Each dose group was compared with the vehicle control group. The calculations were performed and graphed using GraphPad Prism (version 4.03, GraphPad, La Jolla, CA). The AUC was analyzed by a 1-way ANOVA with a Dunnett's post hoc test.

Novel Object Recognition

The animals are habituated to the novel object arenas for one day prior to testing. This is accomplished by placing the animal in the arena with no objects for 10 min and then placed back

into the home cage. The arena is then cleaned with 70% ethanol in between animals. The day of the test the animals are habituated to the dosing room for 2 hours. The animals were administered VU6024578 (1-10 mg/kg p.o.) or vehicle (10% Tween 80) and were placed back into their homecage for 60 min. The animals were then injected with MK-801 (0.075 mg/kg 1 ml/kg s.c.) and placed back in the homecage for 30 min. The animals are then placed into the arena with two identical familiar objects for ten minutes and then placed back into the home cage. The animals are then placed back into the arena with one of the familiar objects replaced with a novel object at 120 min after the familiar object exposure. The activity of the animals are recorded and the time spent exploring each of the objects is scored by a blinded observer. Recognition index is determined by subtracting the time spent with the novel object form the time spent with the familiar object divided by total time spent exploring.

Brain Slice Preparation and Fast Scanning Cyclic Voltammetry:

C57Bl6N mice (10-11 weeks of age) were anesthetized with isoflurane prior to transcardial perfusion with an ice-cold cutting solution containing: 87 mM NaCl, 75 mM Sucrose, 26 mM NaHCO3, 25 mM D-glucose, 7 mM MgSO4, 2.5 mM KCl, 1.25 mM NaH2PO4, 1 mM kynurenic acid and 0.5 mM CaCl2. Slices were recovered in artificial cerebrospinal fluid (aCSF) 126 mM NaCl, 26 mM NaHCO3, 11mM D-glucose, 2.5 mM KCl, 2.45mM CaCl2, 1.25mM NaH2PO4 and 1.2mM MgSO4 which was supplemented with 5mM sodium ascorbate. Slices were recovered for at least 1 hour prior to recording. During recording slices were perfused with aCSF in the absence of ascorbate and presence of 1 mM DhβE to block nAChR-mediated dopamine release. All experiments were performed with brain slices being held at a temperature of 32°C and a flow rate of 2mL/min.

Electrically-evoked dopamine overflow was monitored with carbon fiber electrodes as previously reported (Foster et al. 2014). Briefly, a triangular voltage wave (-400 to +1000 mV at 300 V/sec) was applied to a fresh-cut carbon fiber electrode every 100 msec. Dopamine release was evoked via proximal stimulating electrodes with single pulses (1 msec, 300-600 μA) being delivered every 2.5 min. Currents were acquired using a Clampex9.2 / Digidata 1440A system (Molecular Devices) with a low pass Bessell filter at 10kHz and digitized at 100kHz. Background-subtracted voltammograms served to calibrate the electrodes and identify dopamine as the substance being

released. The best-fit simulation of electrically-evoked dopamine release was found by nonlinear regression. All time-course data are presented as the mean ± SEM for individual time points. The effect of drug was determined by averaging the transient amplitudes following DHPG administration (17.5 - 35 min) either in the absence or presence of mGlu1 PAM. Data were analyzed using a two-tailed Mann-Whitney test, and statistical significance was determined as $p < 0.05$.
